# The consequences of regular methamphetamine use in Tehran: qualitative content analysis

**DOI:** 10.1186/s13011-020-00277-3

**Published:** 2020-05-14

**Authors:** Javad Yoosefi Lebni, Arash Ziapour, Mostafa Qorbani, Fereshteh Baygi, Amin Mirzaei, Omid Safari, Babak Rastegarimehr, Bahar Khosravi, Morteza Mansourian

**Affiliations:** 1 Health Education and Health Promotion, School of Health, Iran University of Medical Sciences, Tehran, Iran; 2 Health Education and Health Promotion, Health Institute, Kermanshah University of Medical Sciences, Kermanshah, Iran; 3 Non-Communicable Diseases Research Center, Alborz University of Medical Sciences, Karaj, Iran; 4 Chronic Diseases Research Center, Endocrinology and Metabolism Population Sciences Institute, Tehran University of Medical Sciences, Tehran, Iran; 5 Center of Maritime Health and Society, Institute of Public Health, University of Southern Denmark, Esbjerg, Denmark; 6 Public Health Department, Ilam University of Medical Sciences, Ilam, Iran; 7 Departments of Pediatrics, School of Medicine, Alborz University of Medical Sciences, Karaj, Iran; 8 Abadan School of Medical Sciences, Abadan, Iran; 9 Master of Women Studies, Shahid Madani University of Azerbaijan, Azerbaijan, Iran; 10 Health Management and Economics Research Center, Iran University of Medical Sciences, Tehran, Iran

**Keywords:** Methamphetamine, Regular users, Consequences, Qualitative study, Tehran

## Abstract

**Background:**

In recent years, methamphetamine use has increased noticeably in Iran, and this can make harmful consequences for the health of individuals and society. Therefore, the study aimed to investigate the consequences of regular methamphetamine use in Tehran**.**

**Methods:**

This study was conducted based on a conventional content analysis approach. Data were collected through observation and in-depth interviews with 20 regular adult users of methamphetamine in Tehran (including 15 males and 5 females). Participants were selected using snowball sampling and purposeful sampling, which continued until data saturation. Guba and Lincoln’s criteria were used to assess the strength of the study.

**Results:**

The extraction of the codes resulted in three main categories: (1)the short-term consequences, consisting of the sub-categories of individual and social consequences, (2) the long-term consequences, consisting of the sub-categories of psychological and physical consequences, high-risk behaviors, severely decayed memory and changes in the eating pattern, and (3) hallucinations and delusions including the sub-categories of visual and auditory hallucinations, persecutory delusions and delusion of having supernatural power.

**Conclusion:**

Regular methamphetamine use may have serious adverse effects on the overall health of individuals. It is therefore highly recommended that educational programs must implement with the use of methamphetamine in the high- risk groups in order to raise awareness and change attitudes about the short and long term consequences. is highly recommended.

## Background

Drug use is one of the most controversial issues in the field of social sciences [1, 2], and dependence on it can be recognized as an illness, and the sociopolitical and health problems worldwide [3]. The drug consumption pattern has recently changed in Iran as the young generation is willing to consume new drugs such as methamphetamine, crack, and heroin [4, 5]. Methamphetamine is a stimulant drug [6], which has become popular in Iran with the name of crystal. It is the most frequently abused substance, and its prevalence in Iran is 5.2% of consumers [7]. Insurance companies are reluctant to pay the costs when they detect a psychiatric problem due to drug usage. Thus, the underreporting of drug use is a real problem [8–10]. The growth of methamphetamine consumption in the world [11] as well as in Iran has become an important problem for the health sector [12–14] at the individual and social levels [15, 16].

Ease of production, uncommon compounds, and different degrees of purity, low cost and high income, easy availability, simple and little needed equipment, the possibility of mass production, and difficulty in identifying laboratories make its trade very lucrative [17]. However, it can cause a lot of dangers, such as explosion, burns, lung burns and even cancer, for manufacturers and those who are near the places of production and exposed to waste [18–20].

The regular methamphetamine use can lead to long-term harmful effects [21]. Ahmad Hatim [15] conducted a study on methamphetamine dependence in Malaysia, and the results showed that the prevalence of psychiatric co-morbidity among these people was 54.4%, the prevalence of suicide was 12.1, and 47.9% of these people had high levels of methamphetamine-induced psychosis.

Petit et al. [11], in their study, found that there was a significant association between methamphetamine addiction and personality disorders, and cardiovascular, pulmonary, infectious, and dental diseases. Sommers et al. conducted a study titled “Methamphetamine use among young adults: health and social consequences” and found that regular methamphetamine use was associated with the consequences of weight loss, seizures, and epilepsy. Besides, many methamphetamine abusers experienced severe psychological symptoms or diseases such as depression, hallucinations, paranoia, and violence [22]. Also, in a systematic review performed by Marshall and Warb, there was a strong association between methamphetamine use and increased depression, psychosis, and suicide mortality risk, but no association was found between methamphetamine use and infectious and dental diseases [23, 24].

The abuse of methamphetamine creates a sense of euphoria and lightness in a person, which is highly addictive. Methamphetamine rapidly enters the brain after taking and causes a sudden secretion of neurotransmitters including, norepinephrine, dopamine, and serotonin. The most important consequences of the use of this substance include obsessive-compulsive disorder [25], fetishism [26], depression [27], antisocial behavior [28, 29], violent behavior [30], movement disorders like Parkinson’s disease [31], low mental health [32], insomnia [33], and risky sexual behavior [34].

Although many studies have been conducted on the side effects of methamphetamine worldwide, most of them have been carried out quantitatively, and experimentally. Crystal meth is produced in more different conditions in Iran [35]. Therefore, it has different effects from those of drugs produced in other countries. Also, since qualitative studies provide a better understanding of phenomena and can explain human beings’ life experiences, interpretations, and perceptions following the cultural and social contexts [36, 37], there is a need for qualitative research in this field. Therefore, this study was conducted based on a qualitative content analysis approach aimed at exploring the consequences of regular methamphetamine use in Tehran.

## Methods

### Design and sample

This study was conducted based on a conventional content analysis approach. Content analysis is a qualitative research method used to analyze the data, and it is a systematic classification and coding technique aimed at a better understanding of the phenomenon under a study [38, 39].

The study population consisted of regular methamphetamine users in Tehran who had a history of methamphetamine use at least 3 months and had not previously used another drug. In-depth interviews and observation were used to collect the data. The first author of the paper only observed and noted the behaviors and physical symptoms of a regular methamphetamine user as a roommate without any intervention. Interviews were conducted by the first and eighth authors. In this study, considering the experience of the first author living with a regular methamphetamine user as a roommate for 5 months, his observations were coded and analyzed to record the behavior of that person. The study inclusion criteria were as follows: (1) regular methamphetamine use at the time of the study, (2) a history of regular methamphetamine use at least 3 months, (3) no history of the psychiatric disorder before methamphetamine use, (4) not being addicted to other drugs before or at the time of the study, and (5) the willingness to participate in the study. Regarding the subject of the research and the difficulty of sample identification, snowball sampling, and purposeful were used.

### Data collection and analysis

In the present study, interviews were conducted with regular methamphetamine users in Tehran until data saturation was reached. Data saturation occurred when interviews were conducted with 20 people, including 15 men and 5 women. The codes obtained from the interviews were repeating, and the researchers ensured that no new codes were forming by continuing the interview. Then the theoretical saturation occurred, and the researcher interrupted the interview process because new information was not obtained by continuing the interview [40]. The duration of the interview was adjusted according to the willingness of the participant to answer the questions, which took an average of 40 min.

At the beginning of each interview, demographic questions were asked, and then the interview continued with questions like: How did you feel when you use methamphetamine for the first time? What were the changes that had occurred in your body, personality, and personal and social behaviors due to short-term methamphetamine use (during the first 6 months)? What interesting things did you experience that you felt didn’t happen to others during regular methamphetamine use? Explain the hallucinations you experienced after regular methamphetamine use. During the interviews, the full attention was paid to the appearance and body language of the participants for more comprehensive information. After each interview, the data were coded and analyzed.

After encoding and summarizing the data based on similarities and differences, the classification of codes was performed. The classes were compared together and ultimately. The themes were extracted by the analysis and interpretation of these data. Graneheim and Lundman’s method was used to analyze the data [38]. In the first step, the whole content of the interview was transcribed into word by word and read several times until a general concept of the text was obtained. Then the text was divided into independent concepts and marked with specific codes. In the next step, the codes were divided into subcategories and classified according to similarities and differences. Finally, the hidden content was found and reported. An example of analysis and coding is given in Table 1.

**Table 1:** An example of code analysis

Categories	Subcategories	Codes	Quotes
Short-term consequences	Individual consequences	Enhancing sexual ability	“The first month I was using crystal my sexual power was very good; I was pleased with myself.”
	Social consequences	Getting more social	“I was always an aloof person. My family always kept on at me about being isolated, but since I used crystal, I was not like that anymore, I got so socialized, and my family is pleased with me.”

Guba and Lincoln’s criteria were used to assess the accuracy and rigor of the present study [41]. Regarding the credibility of the data, the authors had a prolonged engagement with the participants to win their trust and have a better perception of their experiences. In addition, the coding and analyzed results were forwarded to some of the participants to check if the results reflected their opinions and viewpoints (member check). To consider the transferability of the data, the obtained information was examined and approved by three specialists in the field of qualitative research and methamphetamine studies. The process of coding and data analysis was sent to them, and their comments were used to name the categories and sub-categories. Also, a comprehensive description of the subject was provided, and the direct quotations from participants were used.

### Ethics

In this study, ethical considerations were made following the Helsinki Declaration. This study was approved by the Ethics Committee of Iran University of Medical Sciences. All subjects were informed about the study and provided the written informed consent. There was an emphasis on maintaining privacy and respecting the honesty in keeping and delivering the information accurately without mentioning the names of the people. Subsequently, participants were given the right to leave the interview at any time, if they wanted to leave the interview process, and they were promised to have the study results if they want.

## Results

### Sample characteristics

The results showed that the average age of the samples was 27 ± 7.36 years and the average duration of their drug use was 25 ± 25.54 months. Also, the majority of the sample were single and male and had earned a bachelor’s degree (Table 2).

**Table 2:** Demographic information of the samples

NO.	Age	Gender	Duration of drug use	Level of Education	Marriage status
1	25	Male	1 year	Bachelor’s degree	Married
2	22	Male	9 months	High school diploma	Single
3	28	Male	2 years	Bachelor’s degree	Single
4	33	Male	3 years	High school diploma	Married
5	18	Male	3 months	High school diploma	Single
6	30	Male	4 years	Bachelor’s degree	Married
7	20	Male	1 year	High school diploma	Single
8	24	Male	1 year	Unfinished high school	Single
9	28	Male	9 months	Bachelor’s degree	Single
10	32	Male	3 years	Unfinished high school	Single
11	29	Male	2 years	Bachelor’s degree	Single
12	49	Male	10 years	Unfinished high school	Married
13	33	Male	1 year	Master’s degree	Single
14	35	Male	3 years	Master’s degree	Married
15	19	Male	10 months	High school diploma	Single
16	23	Female	1 year	High school diploma	Single
17	21	Female	2 years	Bachelor’s degree	Single
18	28	Female	3 years	Bachelor’s degree	Married
19	18	Female	8 months	Unfinished high school	Single
20	25	Female	2 years	Bachelor’s degree	Married

After analyzing the data, three main categories were identified that presented in Table 3; 1. Short-term consequences, consisting of individual and social consequences, 2. Long-term consequences, consisting of physical, psychological, and behavioral consequences and decayed memory, 3. Illusions, consisting of visual and auditory illusions. Then quotes and other explanations are given.

**Table 3:** Categories, subcategories, and codes

Categories	Subcategories	Codes
Short-term consequences	Individual consequences	Getting euphoric, getting happier, getting stronger, getting more focused, enhancing the sexual ability
	Social consequences	Getting more social, more acceptance in the society, family happiness after the first months of drug use
Long-term consequences	Physical consequences	Decayed and broken teeth, dry mouth, sunken eyes, many wounds on the body especially hands, wounded gums, many pimples (under or over the skin) on the face and chin, sleep decrease, having a noisy sleep, pain, and burning of the throat, getting thin.
	Psychological consequences	Depression, having irrational beliefs about associates, thinking about suicide, getting insouciant about everything, Obsessive-Compulsive Disorder (OCD)
	High-risk behaviors	Doing dangerous activities like high-speed driving, committing suicide, fighting a lot, mistreating the spouse, loss of control of behavior, high levels of violence against associates, wounding their bodies, having sex with multiple people, decrease of sexual power, getting rougher in sex, Jumping from high altitude due to lack of correct distance detection, excessive talking, excessive anger.
	Highly decayed memory	Forgetting words while talking, forgetting the names of relatives, breaking the promise, keeping on at something, missing the addresses many times, losing different things like keys, forgetting the password of the bank card, lack of focus.
	Changes in eating pattern	Excessive consumption of drinks specifically soft drink, lack of tendency to consume sour things like yogurt drink, tendency to overeat sweats, lack of balance in eating, eating less
Hallucinations and delusions	Visual Hallucinations	Seeing strange things, seeing things other than their face in the mirror, the illusion of believing in things that are not real, the illusion of living with imaginary people, having horns on the head in the mirror, seeing someone sitting next to the bed at night, seeing a little girl with long hair, the illusion of seeing the elephant while driving, seeing trees as police officers, the illusion of seeing the sea and the ship, the illusions of hitting people by car (while they hit a cat), seeing small ants on the arm, claiming to see things that others cannot see, and the illusion of attending a big concert
	Auditory Hallucinations	To hear the result of the football game before it starts, to hear a voice encouraging the person to commit suicide, to hear the sound of walking feet in the sleep, to hear strange noises
	Persecutory Delusions	Conspiracy of colleagues to kill them, suspicion about spouse’s betrayal, suspicion about neighbors for the desire to kill them, the illusion of being chased by anonymous people, the illusion of an individual coming to the room and moving the thing, the illusion of being harmed by others and the illusion of cameras being in the houses they rented.
	The delusion of having supernatural power	Believing in having the perfect sixth sense and the illusion of predicting everything (like results of soccer games)

#### Short-term consequences

Initially, regular methamphetamine use has associated with positive outcomes, such as increased concentration and sexual ability, but these positive outcomes are not sustainable, and after some time these positive effects are replaced by the negative outcomes, such as hallucinations, etc.

Consequences that appear to be beneficial which makes it more satisfactory to the person, but they are not sustainable.

#### Individual consequences

Regular methamphetamine use initially causes physical and psychological changes in an individual that can affect his or her behavior, and in some cases, these behaviors are appealing to a person.

Participant No. 7 said: “I got much more focused at the beginning of using the crystal. I studied less, but I learned more, and my grades were much better, I was very pleased with myself.”

Participant No. 1 said: “The first month I was using crystal, my sexual power was very good, I was pleased with myself.” Participant No. 11 also said: “When I started to use crystal, I got stronger, and I lifted weights more than ever.”

#### Social consequences

The short-term consumption of methamphetamine creates self-confidence in the person, and this changes the person’s social behavior. Unfortunately, one of the features of methamphetamine is that it has positive effects on the early short-term period, which causes not to detect bad effects in a short period.

Participant No. 2 said: “I was always an aloof person. My family always kept on at me for being isolated, but since I used crystal, I was not like that anymore, I got so socialized, and my family is pleased with me.”

Participant No. 9 said: “I was afraid of being in the crowd. I did not like to be at parties. If I went, I sat quietly, and do not say anything. But after starting using crystal, I felt more confident, I talk more in parties, and I like more to be with people.”

#### Long-term consequences of methamphetamine consumption

Long-term consequences of methamphetamine consumption are more destructive than those of other substances, both in terms of physical and psychological consequences of this substance and. In fact, after a relatively short period of initial consumption, its harmful effects are observed which may lead to harm to the regular user or their family or even the society and completely disturb the life of the user.

#### Physical consequences

The effects of methamphetamine consumption in long-period cause changes in the body of regular users, which can disrupt their life and endanger the health of the individual.

Participant No. 12 said: “My body always itches; sometimes I scratch my skin so much that it gets wounded and bleeds”. Participant No. 10 also said: “All my teeth are rotten. I can hardly eat anything, whenever I eat my gums bleed”.

Participant No. 4 also said: “I cannot sleep like before; my sleep is messed up; sometimes I cannot sleep at all.”

#### Psychological consequences

Regular methamphetamine use causes mental changes in the regular user, which can disrupt his/her life.

Participant No. 18 said: “After a while using crystal, I got insouciant about everything, I do not care about anything. Nothing makes me happy or sad.” Participant No. 20 said: “For a while, I think about suicide all the time. I think about how to kill myself.” Participant No. 8 said: “I’ve been very obsessive-compulsive recently. When I want to do something, I think about it so much that I cannot do it, and its time passes.”

#### High-risk behaviors

Long-term consumption of methamphetamine causes the regular user to show unpredictable behaviors that can endanger their own lives and others’ lives, and may cause death to themselves or their associates.

Participant No. 6 said: “Recently, I’ve been fighting all the time. I beat my wife. I fight with my in-law family. I keep on at everyone.”

Participant No. 10 said: “I have sex with many people, although I know it can be dangerous, I do it again.” Participant No. 4 said: “I speak angrily to my wife every day; sometimes I beat her so much that I fear that she will die.”

#### Highly decayed memory

Long-term consumption of methamphetamine leads to memory decay so that the person faces difficulty in doing regular activities.

Participant No. 8 said: “I forget my friends’ names; I even confuse the names of my family members.”

Participant No. 12 said: “I got very forgetful lately; I forget my password and give the wrong one.”

Participant No 6 said: “Sometimes I miss my home address even though we have been living there for ten years.”

Participant No. 12 said: “Sometimes it happens that I get myself busy doing something. Then I look at the clock and see that I only sit down for eight or nine hours getting busy with that idle thing.”

#### Changes in the eating pattern

Long-term consumption of methamphetamine causes changes in nutrition patterns of eating and may lead to a tendency towards foods or drinks that were not consumed by them before.

Participant No. 20 said: “I used to love sour things a lot before, but since I started using crystal I do not like them, I’d love to eat sweet things and drink very much, too.” Participant No. 19 said: “Since I started using crystal, my appetite has decreased, and I’ve been eating too little.” Participant No. 13 said: “My eating is not balanced. I sometimes eat so much that my family gets worried. Sometimes it happens that I do not have anything for two days.”

#### Common hallucinations and delusions caused by the consumption of methamphetamine

One of the common consequences of methamphetamine consumption is the creation of hallucinations and delusions in people, which sometimes causes violence against themselves and others, and may even result in murder and suicide.

#### Visual hallucination

Methamphetamine consumption causes visual hallucinations in regular users, such as claiming to see things that others cannot see.

Participant No 3 said: “In an evening that I was driving, I hit a cat with my car, but I thought I hit a person. For a while, I felt I was being chased. I hid.”

Participant No. 10 said: “Sometimes I see tiny ants that march on my arm, so I scratch my arm until they all die.”

Participant No. 16 said: “At night, a little girl comes and sits next to my bed, I say to my family, but they say there is nothing.”

#### Auditory hallucinations

Methamphetamine consumption also results in auditory hallucinations, as many methamphetamine consumers claim to be able to hear voices that others cannot hear.

Participant No. 17 said: “Sometimes a voice keeps on at me that I should kill myself. I tried to kill myself a few times, but my family did not let me.” Participant No. 3 said: “When I sleep, I hear feet that come close to me, but as I open my eyes, I see nobody. But when I close my eyes again, I hear the feet.”

#### Persecutory delusions

Another problem that appears with the regular use of methamphetamine is the persecutory delusions. It makes a person pessimistic about others and may lead to being violent against them.

Participant No 12 said: “I always felt that my wife was betraying me, so I did not let her go out, and she was always under my observation, this caused us to fight, and I always beat her.”

Participant No. 10 said: “I always feel that my colleagues want to kill me, once the butler wanted to put poison in my tea, but I understood it.” Participant No. 7 said: “I feel like everyone wants me to die, they all want to kill me.”

#### Delusion of having supernatural power

Methamphetamine consumption in some cases causes a person to claim to have supernatural powers and have a delusion of being associated with the universe.

Participant No. 9 said: “My sixth sense is perfect, and I can predict everything even the results of football matches, I feel someone comes to me before the game starts and tells me the result of the game.”

Participant No. 11 said: “I can predict the upcoming events; I can even figure out who is about to die.”

## Discussion

This study aimed to explore the consequences of regular methamphetamine use in Tehran. The results showed that methamphetamine consumption in the short period is accompanied by individuals with the consequences of being happier, increased confidence, increased concentration, improved sexual power and social consequences such as getting more sociable, etc., leading to more desire to continue of the consumption, which was in line with results found by Kennedy [42], Bustani and Karamizadeh [43], Mohib Ali et al. [44], and Sia Johnny et al. [45]. However, our results showed that positive outcomes were replaced gradually by negative outcomes over time.

In Baangy et al.’s study, insomnia [32], and in Shakiba et al.’s study, an increase of excitement and concentration [13] have been identified as short-term consequences of methamphetamine consumption.

The positive short-term consequences of methamphetamine may cause the family to be more pleased with the individual, and it encourages the person to continue the use of methamphetamine. Since the families are more satisfied with the change in the person’s behavior, they have less doubt that perhaps this change in behavior is due to methamphetamine consumption. But the regular use of methamphetamine, in the long run, is accompanied by extremely dangerous consequences for the individual and the society, which can disrupt the life of the individual and society.

According to the findings of this study, the physical and psychological consequences such as high-risk behaviors, decayed memory, and changes in the pattern of eating were among the sub-categories related to the consequences of long-term methamphetamine consumption. The results showed that long-term methamphetamine consumption would affect the human body and could have harmful effects on it, endangering the health of the individual and even leading to death. Previous studies reported the association between regular methamphetamine use, and human physical health, for example, the relationship between the methamphetamine abuse, oral dryness(dry mouth), cardiovascular diseases [46], infectious diseases such as endocarditis [47] and teeth decay [48, 49] has been proven in this area.

Psychological consequences such as depression, having irrational beliefs about associates, thinking about suicide, insouciance about everything, and obsessive-compulsive disorder are other effects of the long-term methamphetamine consumption in this study. The results of previous studies showed that the abuse of methamphetamine is associated with the sustainable psychological changes in regular users, such as depression, isolation, personal conflicts [50], irrational thoughts [51], and obsessive-compulsive disorder [28].

Another sub-category related to long-term consequences was high- risk behaviors. Long-term consumption of methamphetamine can change a person’s behavior and increase high-risk behaviors [52]. Changes in behavior and an increase in risky behaviors are the symptoms of long-term regular use of methamphetamine. Violence, aggressiveness, and hostile attitudes are also common in methamphetamine users [53, 54]. Also, regular use of methamphetamine can increase the sexual need of an individual abnormally. Because of the other effects of this substance, it causes incontinence in a person, so that he no longer uses preventive measures and devices. And the person is forced to indulge in sexual activities with multiple people that can endanger the health of the person, leading to AIDS, hepatitis, and other sexually transmitted diseases [55–59].

Another symptom of long-term regular use of methamphetamine is memory disorder. Maxwell (2005) and Lundqvist (2005) found a significant relationship between the methamphetamine abuse and the decreased cognitive and psychosocial functioning in the individual [59, 60], which caused problems such as memory decrease, confusion, and forgetfulness [61, 62]. Continued use can also result in memory loss, and people with it resemble those who have Alzheimer’s disease [63].

Changes in the pattern of eating are another sub-category of long-term consequences, which was a new finding that is not mentioned in other studies. Changes in eating patterns and excessive loss of appetite have to do with using methamphetamine so that regular users lose weight and suffer from malnutrition after a while [64].. However, the consequence of methamphetamine consumption may be attractive to some people. Maybe one of the main reasons for the tendency to methamphetamine, especially for women, is their decision for weight loss. As shown in previous studies, the weight of women who consume methamphetamine is significantly less than that of those who do not use it [65]. The results obtained from the study of Tsirigotis also showed that addicted women experienced more indirect self-destructiveness than their addicted men [66].

Another consequence of regular use of methamphetamine is the hallucination and delusion in the users, which is not obtained from previous studies. These hallucinations and delusions sometimes lead to social harm and murder, which we see a title related to this issue (e.g. Crystal user Killed his Family) in the newspapers every day, and it increases day by day due to the increasing number of methamphetamine consumers. The common visual hallucinations that these people have included seeing things without real existence, and no one else can see these things except them. In some cases, users have the hallucination of seeing insects on their skin, which results in scratching and wounding their skin, which is why many people who are using this drug usually have a lot of wounds [23, 67]. In the studies of Baggott et al. and Akiyama, visual hallucinations caused by methamphetamine use were also reported [68, 69].

Auditory hallucinations are also observed in them, which sometimes stimulate the regular users to hurt themselves and others. Auditory hallucinations were also reported in methamphetamine-dependent in the study of Mahoney et al. [70].

Another form of hallucination and delusion in these people is the delusion of having supernatural power and can fight the whole world. It creates a kind of false self-confidence in them. The majority of regular methamphetamine users believed that they have superhuman strength to do extraordinary things, such as predicting the future, and because these people saw themselves as safe in danger, they took dangerous actions and endangered their health.

But perhaps the worst kind of delusions in these people is the delusion of others’ conspiracy against them [25]. Many regular methamphetamine users experience this delusion that others are thinking about harming them or their wives or husbands or plans to betray them. This type of delusion makes these people commit dangerous unpredictable behaviors. One of the new results in this study, which is not mentioned in previous studies, maybe due to differences in the composition of methamphetamine made of different compounds in Iran and having different effects. Sometimes the severity of these behaviors can be highly bothered for society. The results of the study of Mansourian et al. also showed that methamphetamine users have a suspicion about associates [67].

### Strengths and limitations

This was the first Iranian study that addressed the consequences of regular methamphetamine use in Tehran, the capital city of Iran, employing a qualitative approach since first-hand information can be attained by doing so. Moreover, given that the first author of the present study experienced the lifestyle of his methamphetamine addicted roommate, the real behaviors of a regular methamphetamine user could be identified and recorded easily to some extent. Another strength of the study was the selection of subjects with no history of previous use of any other drugs except methamphetamine, which could help discover the specific consequences of regular methamphetamine use. However, there were some limitations, too. One of the main limitations was the difficulty of access to regular methamphetamine users, which was resolved by choosing an appropriate sampling method and drawing the trust of the samples to introduce their peer user friends. Another problem was determining the time and place of the interview, which was repeatedly changed by the participants. The researchers solved this problem by frequently visiting the places of the interview.

## Conclusion

The harmful consequences of methamphetamine consumption can endanger the health of the individual, family, and society. Therefore, raising the knowledge about the short-term and long-term effects of regular methamphetamine use, its symptoms as well as providing appropriate programs to change attitudes toward the consequences can be an effective step in preventing the use of this drug. Besides, appropriate counseling to regular methamphetamine users and assisting their rehabilitation to return to a healthy life, are recommended to control the adverse effects caused by the use of this drug.

## Data Availability

The datasets generated and analyzed during the current study are not Publicly available due to protect the participants’ anonymity but are Available from the corresponding author on reasonable request.

## Abbreviations

MH: Methamphetamine regular use
QCA: Qualitative Content Analysis
